# 9-1-1 Call Data Modeling to Assess the Influence of a Mass Gathering on Host Community Metrics for Syndromic Surveillance

**DOI:** 10.5811/westjem.53142

**Published:** 2026-06-16

**Authors:** Arthur H. Yancey, Elijah Robinson, Timothy McMahan, George A. Cotsonis

**Affiliations:** * Emory University, School of Medicine, Department of Emergency Medicine, Atlanta, Georgia; † Grady Emergency Medical Services Information Systems, Atlanta, Georgia; ‡ Emory University School of Public Health, Department of Biostatistics, Atlanta, Georgia

## Abstract

**Introduction:**

Multi-day planned mass gatherings are prevalent in various forms of sports competitions, concerts, professional, political, and religious conferences. The largest, the World Cup competition, will be hosted by large cities of the United States, Canada, and Mexico in June and July, 2026. These can present risks to the health of spectators, participants, organizers, and host communities, which if discovered faster, facilitates more effective responses in preventing morbidity/mortality. This study demonstrates visually modeled 9-1-1 medical data that uniquely contributes to existing syndromic surveillance activities by its practicality and time sensitivity.

**Objectives:**

Using data from the nine-day period of 2019 Super Bowl LIII festivities, this retrospective, quantitative, descriptive, population-based study was conducted to demonstrate how 9-1-1 emergency medical dispatch (EMD) data could be practically modeled to more rapidly detect changes from baseline host community 9-1-1 data. Our ultimate objective is to illustrate these visual models in the attempt to improve syndromic surveillance.

**Methods:**

All 9-1-1 medical calls from three host municipalities were processed and categorized at the Grady Emergency Medical Services (EMS) Communications Center during game day and eight preceding days of festivities (time frame-standardized to nine-day events period). This events period call volume was contrasted to that of the identical nine-day pre- and post-events periods. The mean 24 one-hour interval call volumes for each day of these three periods were analyzed. Data visualization techniques (a data table, bar graphs, geo-, and heat-mapping) were constructed to illustrate differences in 9-1-1 call volumes of three categories (hypothermia, traffic injury incidents, and violence-induced injuries), for the three periods. The most similar previous such studies were reviewed and related to our study.

**Results:**

The events period call volume (3,267) was less than that in pre- (3,356) and post-(3,460) events totals. Directional (increasing versus decreasing) patterns in hourly call frequencies were similar among all three periods. Cold exposure calls decreased across the three periods (successive period were 9, 7, and 4 respectively). Changes in traffic incidents were minimal (36, 39, and 38, respectively). Violent act injury calls increased during the post-events period (151, 150, and 166, respectively).

**Conclusion:**

Data from 9-1-1 calls could provide readily discoverable trends reflective of clinically significant syndromes threatening host community health status associated with planned mass gatherings. The study demonstrates practical data modeling to enhance syndromic surveillance. Recommendations are offered for enhancing 9-1-1 data analysis in conducting syndromic surveillance.

## INTRODUCTION

The 2019 Super Bowl LIII and its multi-day, pre-game festivities, which were held in the City of Atlanta, Georgia and surrounding municipalities, presented the opportunity to assess the feasibility of using 9-1-1 emergency medical dispatch (EMD) data for improving syndromic surveillance of planned mass gatherings and their host communities. Syndromic surveillance includes public health-related activities that involve data collection and analyses to rapidly identify and, if necessary, respond to injury and disease outbreaks.^1^

Although the Super Bowl championship game was played in Atlanta, there is a large event-related entertainment venue in the City of College Park. Furthermore, the necessity of traversing a contiguous third municipality (East Point) to reach College Park from Atlanta provided the rationale for its study inclusion. At the time of Super Bowl LIII, 540,000 citizens of, and visitors to, these municipalities were potentially exposed to and could have been affected by health threats associated with the events festivities. All 9-1-1 medical calls from these three municipalities are routed to the Grady EMS (GEMS) Emergency Communications Center for standardized processing through the EMD call-processing software program.

The Super Bowl LIII Game and pre-game festivities were hosted across a nine-day period in the late January–February 2019. Concerts, festivals, parties, awards ceremonies, military aerial exhibitions, permitted rallies, and protests were held preceding the championship game. According to a media-based report, “more than one-half million people went to Super Bowl Experience and Super Bowl Live.”^2^ There were no known official estimates of attendance at > 100 related overlapping events held at various times across the nine days,^2^ Following this multi-day, multi-festivity mass gathering, the host cities’ 9-1-1 EMD total call volumes, selected specific types, call-time periodicity, and geographic patterns of call origin were investigated to search for data patterns that could have represented threats to community health status. The resulting data were modeled for practical vision-based analysis, in a search for possible roles in conducting mass gathering syndromic surveillance.

The near real-time analysis of 9-1-1 EMD data can provide unique potential contributions to public health surveillance. The term, “practical significance,” refers to variations in events and/or comparison period call volumes that are large enough to threaten or actually deplete community healthcare resource availabilities.The immediate and continual access to data, as events evolve, could lead to earlier discovery of health threats. This should enhance mobile response resource re-positioning, future strategic resource planning, as well as etiology investigation for syndrome mitigation.^3^ These characteristics are critical to improving life-saving response measures.

Published investigations pertaining to use of EMD data for enhancing population-based syndromic surveillance for mass gatherings are few and nonevidence based. Only two are Super Bowl-based. These provide documentation of surveillance resources from which morbidity data was collected. No host community-based EMD data was provided. Plans for collecting this data was mentioned in one study.^4^ A retrospective Canadian study covering public health surveillance of the 2018 G7 Summit reported no increases or differences in specified call types into Info-Sante. Pre-selected categories of clinical call type were listed without corresponding call volume data.^5^ A scoping Dutch review of combined utility of emergency call center, dispatch, and ambulance data was executed for syndromic surveillance limited to infectious diseases, and not focused solely on or mainly for mass gatherings.^6^

**Population Health Research Capsule**

What do we already know about this issue?

*Research is sparse regarding the potential contribution of 9-1-1 call data to syndromic surveillance in the setting of mass gatherings*.

What was the research question?

*Can modeling be designed to display evolving statistics for timely detection of threatening health trends?*

What was the major finding of the study?

*Data are modeled in a table, bar graphs, a time-period graph, geographic point, and heat maps*.

How does this improve population health?

*9-1-1 call data could be used to detect and track health threats as they relate to their boundary areas, direction, speed, and density*.

Conclusions drawn by interviewing participating experts were that EMD surveillance timeliness was most advantageous, followed by cost efficiency, convenience, and data standardization. The public health implication in these articles is that 9-1-1 EMD data analysis may enhance other more traditional, less time-sensitive sources and modes of public health surveillance by contributing the aforementioned attributes. Our study uniquely displays modeling of this readily generated, easily visible, medically standardized and categorized call volume data, to rapidly facilitate discovery of threatening trends, their medical nature, and geographic traits of size, density, rate and direction of spread. Our display of real call volume statistics also reveals the visibly minimal differences that categorical comparisons illustrate during uneventful baseline periods.

The intent of this study is to demonstrate the transformation of 9-1-1 medical calls data from this multi-day mass gathering and its host communities into visible surveillance formats that are easily comprehended, and then describe the qualitative analysis contrasting results in three distinct standardized time frames of nine days each: before (pre-events); during (events); and after (post-events). The subsequent discussion includes recommendations by which visible modeling of this data may enhance syndromic surveillance in the service of mass gathering and host community health protection.

## METHODS

### Data Source and Collection

This retrospective study is based on secondary data collected by GEMS EMD call processing. The Priority Dispatch Corp.’s International Academy of Emergency Dispatch (IAED), Medical Priority Dispatch System (MPDS) software protocols are used by GEMS’ communication center personnel to process each 9-1-1 medical call.^7^ The callers answer telecommunicator-directed questions from the standardized MPDS V13.1 protocols. Answers are classified using IAED-designed electronic classification of answers. For example, all call questions related to cold exposure are listed in protocol 20. The corresponding answers are translated into programmatically matched standardized category codes that identify standardized sets of response resources to be sent to patient locations, thereby, optimizing system effectiveness and efficiency. Telecommunicators simultaneously provide the callers with standardized scripted instructions for efficient bystander-provided patient care appropriate to the telephonic setting. No duplicate calls are included in the reported call volumes, as digital call batching through electronic capture of caller location/address and confirmatory call processing eliminates recurrent calls about the same incident. “False alarms” are never classified as such through 9-1-1 calls, and a physical response to the caller location is dispatched for disposition and EMS classification. The data resulting from processing these calls was categorized digitally by MPDS v13.1 at GEMS, through use of Microsoft Access.

### Organization of Study Population Data

The assessment of the event’s effect on 9-1-1 call demand for EMS services was designed and organized as follows. The total medical call volume during the nine-day events period (01/26/2019 – 02/03/2019) was contrasted with those of the nine-day pre- (01/17/2019 – 01/25/2019) and nine-day post- (02/04/2019 – 02/12/2019) events periods. The study time frames were unified according to the nine days (events period) that encompassed all Super Bowl-related activities. Methods for more detailed investigation of the temporal effects of events period festivities on call volumes were conducted by analyses of the collated 24 one-hour interval 9-1-1 call volumes for each day of the events period compared to those same one-hour intervals of the daily pre- and post-events periods.

The following three specific medical emergency types from four MPDS v13.1 software protocols were selected, based upon the theoretical likelihood of their increased rates during the events period, and more important to the integrity of the study, linkage to the likelihood of their timely optimal initial discovery and thorough data documentation by way of 9-1-1 call capture and processing.

1. Hypothermia (MPDS Protocol 20) from cold exposure was included because most event activities were held outdoors at this winter event. The average minimum daily temperatures in each period were collected for correlation with period-based hypothermia call volumes.
2. Traffic/transportation injuries (MPDS Protocol 29) were included in anticipation of a concentrated influx of motor vehicles and vehicle-pedestrian interfaces in the ambulatory population-concentrated central portion of the city, where most outdoor festivities were held.
3. Violence, including stabs/gunshots (MPDS Protocol 27), and assault (MPDS Protocol 4) categories, was included in anticipation of a social environment of tense emotions from festivities-related revelry and competition rivalry^8^.

### Data Visualization Designs

Data were summarized in a table and plotted in the form of bar charts to facilitate formation of visual impressions of contrasts between total call volumes in the events period and the baseline comparison (pre- and post-events) periods. Since the data collected represented emergency care demand from the entire stable population of the three host municipalities, statistical tests and confidence intervals are not appropriate or necessary. Data presented here are descriptive and should not be used to infer results at any other event. The data analysis was conducted to search for contrasting meaningful differences among the three periods. The practical significance used refers to results large enough to matter in real world applications. In this study, such significance was reached only if increase(s) in 9-1-1 call-mediated demand for emergency clinical care resources occurred to a degree that it threatened to deplete, or actually depleted, the availability of routinely assigned community EMS resources.

Two mapping techniques were applied to the data to detect incident clustering. Heat mapping was used to contextually visualize the total 9-1-1 call volume geographic densities for all three periods.^9^ With this technique, each mapped incident serves as the center of a radius that expands to areas of overlap with its neighboring incidents. Through degrees of color shading, each neighboring incident, therefore, had a contextualized visual relationship with all other neighbors, such that the heatmapping provided immediate visual impression information on call location density trends.

Geographic point-mapping of each of the three types of categorized 9-1-1 calls (ArcMap 10.7.1 software), can include measures of call location spacing, with use of a distance scale. This technique can facilitate formation of traceable patterns of density through display of comparative syndrome progression/regression of incidents across time by sequentially re-mapping evolving data with the same template. Practically significant differences throughout the comparisons of period data groups refer to whether EMS resources designed and purposed for the routine level out-of-hospital care for the public were threatened with depletion by a surge or surges in 9-1-1 call demand volume and/or call concentration beyond the capacity for a host jurisdiction to provide them.

The Emory University Institutional Review Board deemed this study exempt from IRB review, because it was “not human subjects research.”

## RESULTS

### Call Demand and Time Distribution

**Figure 1:**
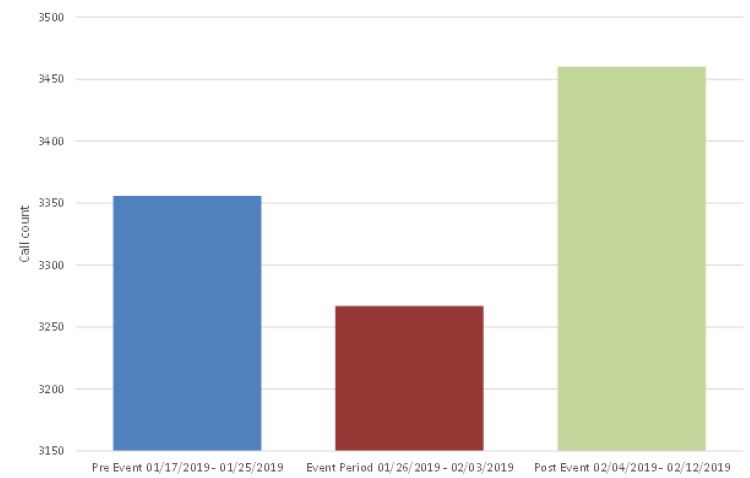
9-1-1 medical calls of the 9-day pre-event, event, and post-event periods for the evaluation of the study mass gathering’s influence on the host community’s total call volume.

Total call volume during the events period decreased relative to the pre- and post-events periods (Figure 1).

**Figure 2:**
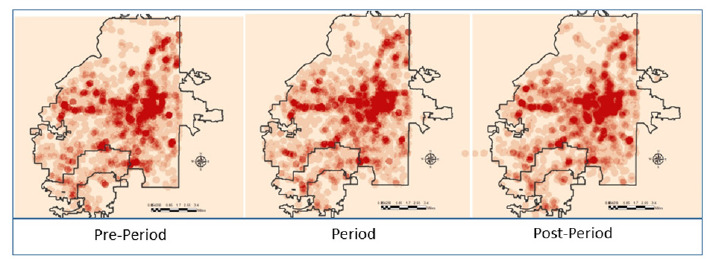
Heat maps illustrating densities of total 9-1-1 medical call locations for the pre-event, event, and post-event periods to evaluate the influence of the study mass gathering on the host community’s call volume.

Heat maps did not reveal visible differences in call volumes or geographic patterns of call concentrations among pre-events, events, and post-events periods (Figure 2).

**Figure 3:**
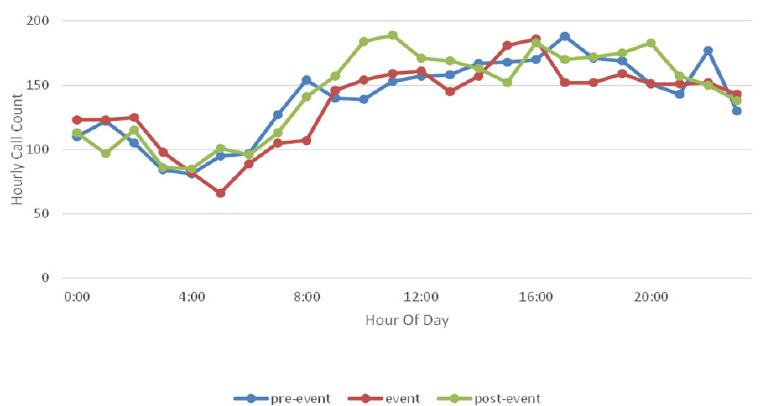
Hourly mean 9-1-1 medical call counts for the pre-event, event, and post-event periods to evaluate the influence of the study mass gathering on the host community’s call volume.

The total hourly call-demand counts revealed similar trends in the three periods, throughout the 24 one-hour time frames (Figure 3).

The highest one-hour interval total of 9-1-1 medical calls in the nine-day events period occurred from 3 pm – 4 pm. This total did not exceed either of the peaks in the two comparison periods in any one-hour interval. The lowest one-hour call volume was in the events period from 4 am – 5 am.

### Call Types

**Figure 4:**
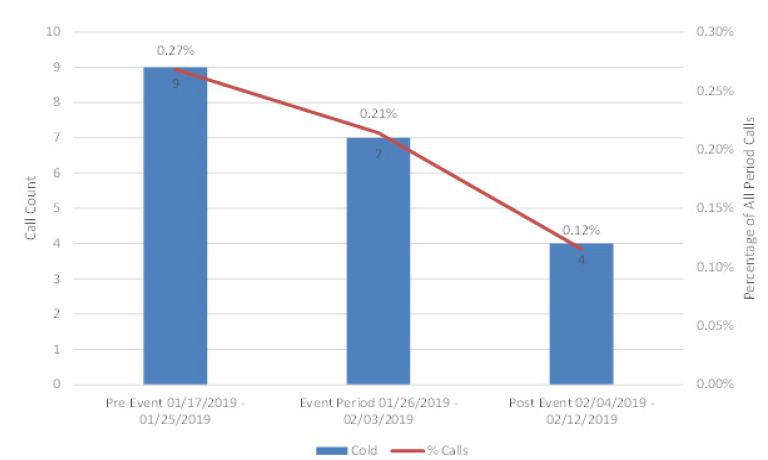
Cold exposure 9-1-1 medical calls for the pre-event, event, and post-event periods including number of calls and percent of these calls for each period to evaluate the influence of the study mass gathering on the host community’s call volume.

Cold exposure injury calls analysis illustrates the declining volumes of 9-1-1 calls in the three successive study periods (Figure 4).

The average minimum daily temperatures were 35.78 °F. in the pre-events period, 33.11 in the events period, and 46.78 in the post-events period, with no meaningful differences in period call volumes that correlated with the minimal temperatures among the three periods.

**Figure 5:**
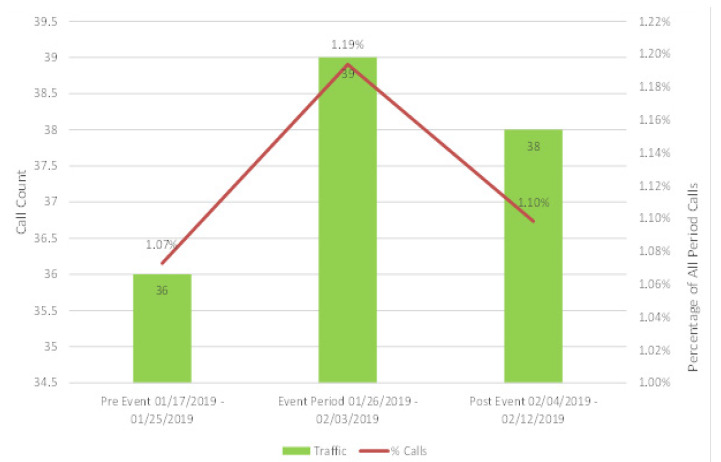
Traffic/transportation 9-1-1 medical calls for pre-event, event, and post-event periods including number of calls and percent of these calls for each period to evaluate the influence of the study mass gathering on the host community’s call volume.

The number of injury-related traffic incidents in the pre-events period was lower than those in the events and post-events periods (Figure 5).

The increase during the events period, remained in the post-events period. The subset of pedestrian vs. vehicle crashes and their subsequent related 9-1-1 calls eradicated the minimal variation in the preceding pattern (Table 1).

**Figure 6:**
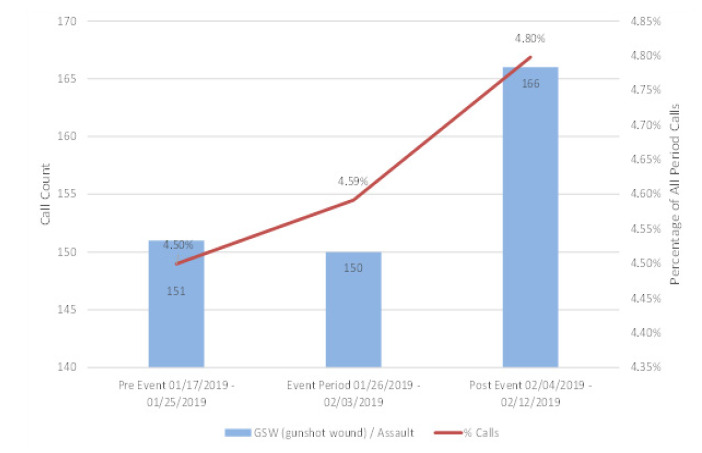
Violent incident 9-1-1 medical calls for the pre-event, event, and post-event periods, including number of calls and percent of these calls for each period, to evaluate the possible influence of the study mass gathering on the host community’s call volume.

Violent acts, resulting in traumatic injury-related 9-1-1 calls, revealed marked similarity between the pre-events period and the events period. (Figure 6).

**Figure 7:**
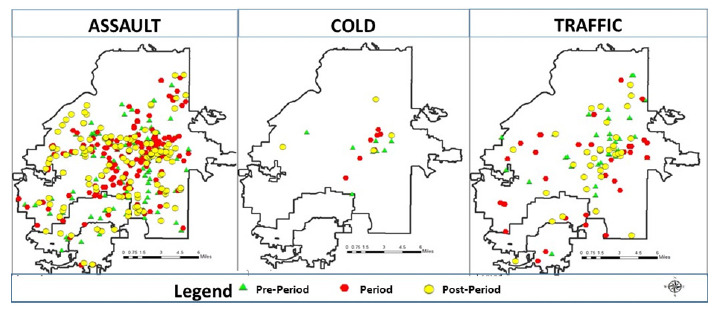
ArcMap 10.7.1 geographical maps illustrating 9-1-1 cold, traffic, and assault injury call locations across the pre-event, event, and post-event periods to evaluate the influence of the study mass gathering on the host community’s density of type-specific call volume.

An increase was evident in the post-events period, compared to the two preceding periods. Self-inflicted incidents by stab and gunshot were practically insignificant when compared to each period’s categorical numbers (Table 1), and they did not change the preceding trend when these were extracted from totals in each of the three violent trauma time periods. Point geo-mapping of the foregoing three categories in each of the three time periods did not illustrate changes in period density patterns (Figure 7).

## DISCUSSION

### Study Interpretations

This study demonstrated how 9-1-1 EMD data could be displayed for efficiently surveying the host community’s evolving emergency health status and how these corresponding displays could lead to earlier discovery of time-sensitive threats than traditional methods of surveillance, if both were compared to baseline health status. Further studies based on the following data classifications and visual depictions hold important implications for improving mass gathering EMS surveillance planning.

Although non-specific in pertaining to medical information, unexplained total call volume expansion could be crucial and easiest to discover in first alerting respective telecommunications, medical, and public health officials of the need to initiate more detailed investigation of etiology. No meaningful (practical) differences in the call volumes’ peaks and valleys among this study’s events periods were found. The similar trends of events’ and comparison periods’ hourly call volume dipping lowest at the 5 am period could be accounted for by visitors and citizens alike being indoors, asleep, and at low risk for sustaining emergencies. Similarly, the hourly call volume during the events period peaked at 4 pm, equaling the similar trend in the post-events period, and exceeding that in the pre-events comparison period. This peak possibly reflects the time of maximal movement of pedestrian and vehicular traffic in the event period, corresponding to the onset of afternoon vehicular “rush hour,” as well as that of social interactions involving ambulatory event visitors leaving daytime festivities, in transition to subsequent evening festivities, leaving them susceptible to pedestrian-vehicular interface incidents.

Multiple festivities, occasionally simultaneous, occurred throughout the events period. This hourly data measure is valuable in revealing a more granular temporal pattern of tracking related medical emergencies than the daily volumes, facilitating associations of 9-1-1 hourly incident patterns with scheduled and unscheduled event activities. The logical actionable responses to such linkable patterns would be the provision of safety information and instructions to the public about associated risky time periods and their associated possible dangers. The optimal prepositioning of resources and professional responders for injury prevention and time-sensitive responses should also exhibit improvement. Contingency planning for future similar events should include detection measures from 9-1-1 medical call volume monitoring, as depicted herein.

Cold exposure injuries are a prominent possibility at this annual event, given the usual cold weather in the greater Atlanta area and most Super Bowl host communities during their winter season. The decreased volume of cold exposure-related calls is possibly reflective of effective sheltering and clothing provision in reaction to the near- but above-freezing temperatures in the pre-events and events periods, and a warming trend in the post-events period. The extremely small call numbers in each of the three categories prevent declaration of trends of population importance.

The 9-1-1 calls in the traffic/transportation events period superseded that in the pre-events period by three calls, which was of no practical consequence to the host population’s collective health status. The absence of a subsequent decrease in traffic/transportation calls in the post-events period suggests that the occurrence of Super Bowl LIII did not contribute to an increased risk of motor vehicle crashes. The subset of 9-1-1 calls for pedestrian versus vehicle traffic incidents (Table 1) showed no meaningful difference throughout the three periods of study. Comparative dampening of the increase in traffic incidents during the crowded events period was possibly related to increased congestion preventing comparative high speeds, an increased law enforcement presence, and/or street traffic blockages during the events period, reducing speeding and distracted driving.

Tension related to passionate team loyalty and associated rivalry, as well as gambling, is thought to be associated with violence surrounding various sporting events.^8^ An increase in the calls reporting aggregated assaults, gunshots, stabbings, and self-inflicted injuries was documented in the post-events period, compared to both previous periods. Subtracting self-inflicted stabs and gunshots did not erase this pattern of increase (Table 1). This increased frequency suggests the need for future use of special event law enforcement protocols, implemented across study periods to guide investigations of events-related (versus non-events related) causes.

The study did not reveal study periods evidence of adverse events of a magnitude that would threaten depletion of community response resources. Table 1 and Figures 4, 5, and 6 reflect call volumes of specific types and their small percentages (between 0.21%–4.80%) of all calls within their time periods.

### Studies on the Role of 9-1-1 Call Data in Syndromic Surveillance

The organizational structure of the study data visualization models of the foregoing results uniquely incentivizes focus on the issue of 9-1-1 medical data’s role in potential contributions to community syndromic surveillance during planned mass gatherings. The following related publications are few, with either focus on the relationship of 9-1-1 data to syndromic surveillance or the relationship of syndromic surveillance to mass gatherings, but none of them describing and evaluating the role of 9-1-1 data in syndromic surveillance for mass gatherings, as in the present study. A pioneering study of the relationship of using 9-1-1 calls to track influenza-like illness was implemented in New York City by the health department, in 1998^1.0^ This evolved into an investigation of the dispatch center data utility for detecting bioterrorism events that manifest with a respiratory component, such as the inhalational anthrax attacks of 2001. A thorough 2022 scoping review of global scale was executed covering syndromic surveillance systems for mass gatherings, investigating their methodologies (data collection and related analysis) and applications (locations, dates, surveillance periods, event types, data sources, and attendance).^11^ Of the 19 studies reviewed for inclusion in this publication’s data extraction, only one used remote access (mobile phone app), without remote systematic public access to emergency care (EMD).

An expanded scope of public health surveillance for Super Bowl XLI in 2007 involved linking four event-related health departments for sharing their data across a system, known as Early Notification of Community-based Epidemics (ESSENCE).^12^ While 9-1-1 medical data was not used, it could have provided enhanced surveillance data input across these departments, which were representative of both host and hotel guest populations. It would require that a corresponding common or similar emergency medical dispatch system be employed across these four jurisdictions’ 9-1-1 centers, with data feeds into the respective health departments. The data from EMD feeds could have been analyzed for surveillance relevance, then integrated into ESSENCE with results from other data sources.

A 2017 paper from the U.S. Centers for Disease Control and Prevention focused on recommendations for syndromic surveillance systems, specifically in relation to mass gatherings.^13^ The article’s hallmarks of desirable data systems are sustainability and operational continuity, as opposed to “drop-in” surveillance systems, erected only for the span of the event. This is critically important advice for the detection of infectious disease outbreaks, given consideration of prolonged incubation periods. The economic and labor-saving advantages, and the system improvements inherent in using a critical continually operational public safety system such as 9-1-1 were highlighted. Clinical specificity of electronic syndromic surveillance systems could be enhanced by integrating complementary clinical systems, such as EMS patient care reports from on-scene care, toxicology findings, and emergency department reporting. Being able to access and query historical baseline electronic 9-1-1 data prior to mass gathering events is recommended. Recognition that morbidities characterized by high criticality are usually time sensitive (cardiac arrest), and/or obviously visible (injuries), are more accurately captured by 9-1-1 than the subtleties of infectious disease. This data access concept led to the selection of the specific call types in our current study. Regarding data interpretation, this article highlights that longer event durations enrich syndrome identification accuracy. Choice of the multiday Atlanta Super Bowl mass gathering footprint for our study was in recognition of this attribute.

Only the following three peer-reviewed articles addressed the three constructs of 1) emergency medical call data, 2) mass gatherings, and 3) syndromic surveillance, thereby being directly relevant to our evidence-based study. None of these studies provided the 9-1-1 call volumes involved (reflecting degree of demand) from their covered events and/or host communities, and none provided graphic visual data modeling and/or graphic depictions as in this current study.

One relevant publication, based on the earlier 2015 Super Bowl XLIX planning, was a description of the public health surveillance strategies planned.^4^ Sources of data collection were guest hotels, animal control agencies, poison centers, the medical examiner’s office, urgent care facilities, emergency departments, and EMS documentation of on-scene responses. All the data from these various point sources were subsequently reported to the Maricopa County Department of Public Health for centralized integration and pattern analysis every 24 hours. In our present study, the 9-1-1 capture of data and its EMD coding provides the opportunity for superior temporal sensitivity, due to real-time continual electronic collection in a templated fashion, supported by immediately available contrasting historical digital data. Calls into 9-1-1 provide verbal and/or electronic capture of caller location, contributing to the immediate formation of electronic geographic mapping.

The study most closely resembling the structure and methodology of our present one was performed in Quebec, Canada, during the June 2018 G7 Summit.^5^ A prospective strategy for the detection of public health threats was implemented across three days prior, three days during, and six days post-event. No hazardous incidents of a scale that threatened the public’s health were detected. The project data included using that from Info-Sante (Quebec 9-1-1” equivalent). The authors surmised that this type of data would support high sensitivity but low clinical specificity. This would be a credible concern if surveillance emanated solely from 9-1-1 EMD data.

A thorough scoping review of the utility of emergency call center-dispatch data linked to EMS on-scene data for syndromic surveillance of infectious diseases was performed in 2019, derived from twenty peer-reviewed and 24 gray literature publications, as well as 11 interviews of experts.^6^ Conclusions drawn that advocated for employing EMD data in syndromic surveillance were its near real-time data accessibility, high-level data standardization, its clinical nature, and low service cost. While this study was not data-based, these advantages of EMD data were interpreted as being boosted by their combination with EMS on-scene data, collected by field medic evaluations, and documented with digital patient care report tablets. This combination enhances clinical specificity, which 9-1-1 data sources alone may comparatively lack.

The system that has performed continuously operational, digital, early warning bio surveillance for EMS services based on EMD data, is FirstWatch, since 1998.^14^ It has experience in organizing and operating systems for syndromic surveillance of mass gatherings, such as Super Bowl XLVII, as a component of comprehensive long-term surveillance, covering coordination of public health and safety, fire and EMS data from 9-1-1. The implementation of this strategy has provided the program with its broad capabilities to track wide-ranging community morbidities, from disease epidemics, and their more prolonged periods of presentation to drug overdoses, which have prolonged sequences of sudden recurrences, and toxic exposures, both intentional and non-intentional. The outcome of this ongoing tracking is digital historical data that is readily available and prospectively organized for robust timely statistical comparison to the templated data being surveyed during any given mass gatherings. For the XLVII Super Bowl, FirstWatch comprehensively organized this function into “geo-fence” areas that included zones (hospitality, hotel, and convention Center) officially covering the Super Bowl festivities and participants, as well as a citywide zone. Simultaneously, “triggers” were monitored for specific types of classified calls, as in our current study. The call volumes data from the simultaneous Super Bowl/Mardi Gras organizational event strategy and other mass gatherings (Republican and Democratic Party conventions) has not been subcategorized and reported formally which would enable comparison with that from our 2019 Super Bowl LIII.

### Advantages to 9-1-1 Data Use in Syndromic Surveillance

The public has immediate direct access to and interface with 9-1-1 telecommunicators. The 9-1-1 centers’ continual operation enables immediate access to historical digital call-based categorized data. This contributes to shorter time frames for investigations and conduct of on-going uninterrupted analysis. The data is organized and archived according to a standardized emergency medical framework, which facilitates the accuracy and guidance of continual and sequential investigations. Immediate electronic capture of ill or injured individuals’ locations at the time of their inbound 9-1-1 calls enables timely sequential geographic mapping of etiologically related call volumes and selected specific call types. The visual depictions facilitate discovery of syndromic location(s), geographic scope, density of victims, direction and rate of spread, and etiology. These foregoing attributes would be expected to enhance investigations, interventions, and resolution of public safety and health threats by facilitating earlier dispatch of incident-appropriate professionals, in numbers more efficiently appropriate to population need.

### Recommendations for the Enhancement of 9-1-1 Data-based Syndromic Surveillance

The relative deficiency of clinical specificity would qualify as an important limiting characteristic if syndromic surveillance was conducted by sole-source 9-1-1 data analysis. However, continually dedicated inclusion of EMS physicians in EMD call center quality improvement/assurance processes should enhance addressing this issue. The EMD data should be integrated electronically with other, more traditionally sourced syndromic surveillance data and jointly analyzed by EMS clinicians and public health epidemiologists, using the visual surveillance tools depicted in this present study. Concurrent with any given event, real-time reciprocal voice communications should link jurisdictional public health personnel to 9-1-1 telecommunicator staff and EMD/EMS medical directors. This would likely expedite earlier identification of deleterious syndromes, with enhanced clinical accuracy, leading to more appropriate responses. For example, earlier interventions may involve pre-established public address system and media messaging to warn and protect threatened populations, both at hosted mass gatherings and in their surrounding threatened communities.

## LIMITATIONS

This retrospective study used secondary data and did not include pre-formed protocols that could have supported on-scene investigations into possible causal relationships of specific 9-1-1 call incidents to the mass gathering under study. No known attempts were made to obtain concurrent event period-related baseline (denominator) population estimates, as citizens and visitors attended multiple festivities simultaneously, ingressing and egressing the activity areas at unmeasured and highly variable times and dates. Because of this lack of attendance/participation measurement, no attempts to determine denominator data were performed and no incidence rates could be calculated. While other population morbidities were considered for inclusion, respiratory complaints would be problematic in terms of the correlation of 9-1-1 calls to the relatively short events period onset and confounding unrelated influenza season-related calls. The local weather variable’s possible effects on traffic incidents and violence call volumes were not included, as temperature variations were small and climate conditions not considered hazardous throughout all of our study periods.

## CONCLUSION

This was a study on the use of 9-1-1 medical calls data to determine the possible effects of a multi-day mass gathering on the surrounding community’s health status. It presents a process for data collection, organization, and visualization methods tha,t hopefully, will be developed to reveal associated clinical syndromes of a scale and time frame that could threaten or adversely impact event participants’ and the hosting public’s health status. Our retrospective analysis of this call data revealed no adverse event(s) that threatened the health status of the host community. Recommendations are made for enhancing syndromic surveillance from the 9-1-1 medical data domain, to serve the safety and health of mass gathering participants, spectators, and host communities.

## Figures and Tables

**Table 1:** Call volume categories of the nine-day pre-event, event, and post-event periods include undifferentiated totals, cold exposure, traffic collision/transportation incidents, and violence/intentional injuries, in a study that evaluated the extent to which these morbidities adversely affected the host community through call volume expansion. Number of calls and percentage of totals are reported.

Categorical Call Volumes	Pre - Event	Event	Post - Event
Undifferentiated totals	3,356	3,267	3,460
Cold exposure	9 (0.27%)	7 (0.21%)	4 (0.12%)
Traffic collision/Transportation incident	36 (1.07%)	39 (1.19%)	38 (1.10%)
Pedestrians impacted by vehicle (% of all Traffic collison/Transportation incident)	3 (8.30%)	5 (12.80%)	12 (31.60%)
Violence/ Intentional Injuries	151 (4.50%)	150 (4.59%)	166 (4.80%)
Self-inflicted (% of all Violence/ Intentional injuries)	2 (1.30%)	2 (1.30%)	1 (0.60%)
